# Supplementary material for: Imbalanced social-communicative and restricted repetitive behavior subtypes of autism spectrum disorder exhibit different neural circuitry
Source: Commun Biol. 2021 May 14;4:574. doi: 10.1038/s42003-021-02015-2 (PMC8121854; doi:10.1038/s42003-021-02015-2)
Supplement: Supplementary file 1 — Supplementary Material [file 42003_2021_2015_MOESM1_ESM.pdf]

Supplementary Information

Title: Imbalanced social-communicative and restricted repetitive behavior subtypes of autism spectrum disorder exhibit different neural circuitry

Authors: Natasha Bertelsen, Isotta Landi, Richard A. I. Bethlehem, Jakob Seidlitz, Elena Maria Busuoli, Veronica Mandelli, Eleonora Satta, Stavros Trakoshis, Bonnie Auyeung, Prantik Kundu, Eva Loth, Guillaume Dumas, Sarah Baumeister, Christian F. Beckmann, Sven Bölte, Thomas Bourgeron, Tony Charman, Sarah Durston, Christine Ecker, Rosemary J. Holt, Mark H. Johnson, Emily J. H. Jones, Luke Mason, Andreas Meyer-Lindenberg, Carolin Moessnang, Marianne Oldehinkel, Antonio Persico, Julian Tillmann, Steven C. R., Williams, Will Spooren, Declan G. M. Murphy, Jan K. Buitelaar, the EU-AIMS LEAP group, Simon Baron-Cohen, Meng-Chuan Lai, & Michael V. Lombardo

Supplementary Figures

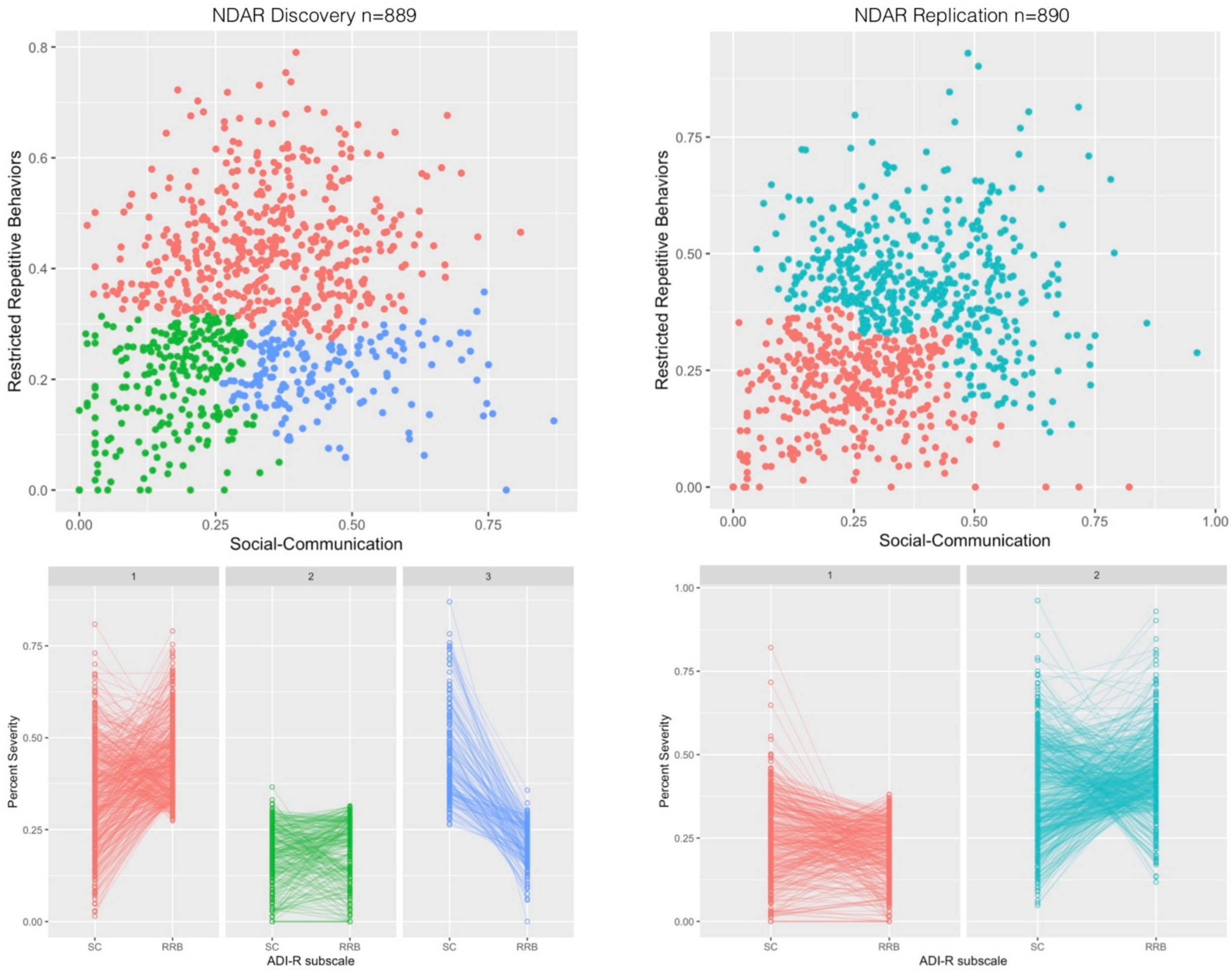

Supplementary Figure 1: Unsupervised stratification with agglomerative hierarchical clustering with static cut height and internal clustering metrics to determine optimal number of clusters. *This figure shows clustering results from NDAR Discovery and Replication datasets when the optimal number of clusters is selected by majority vote with NbClust.*

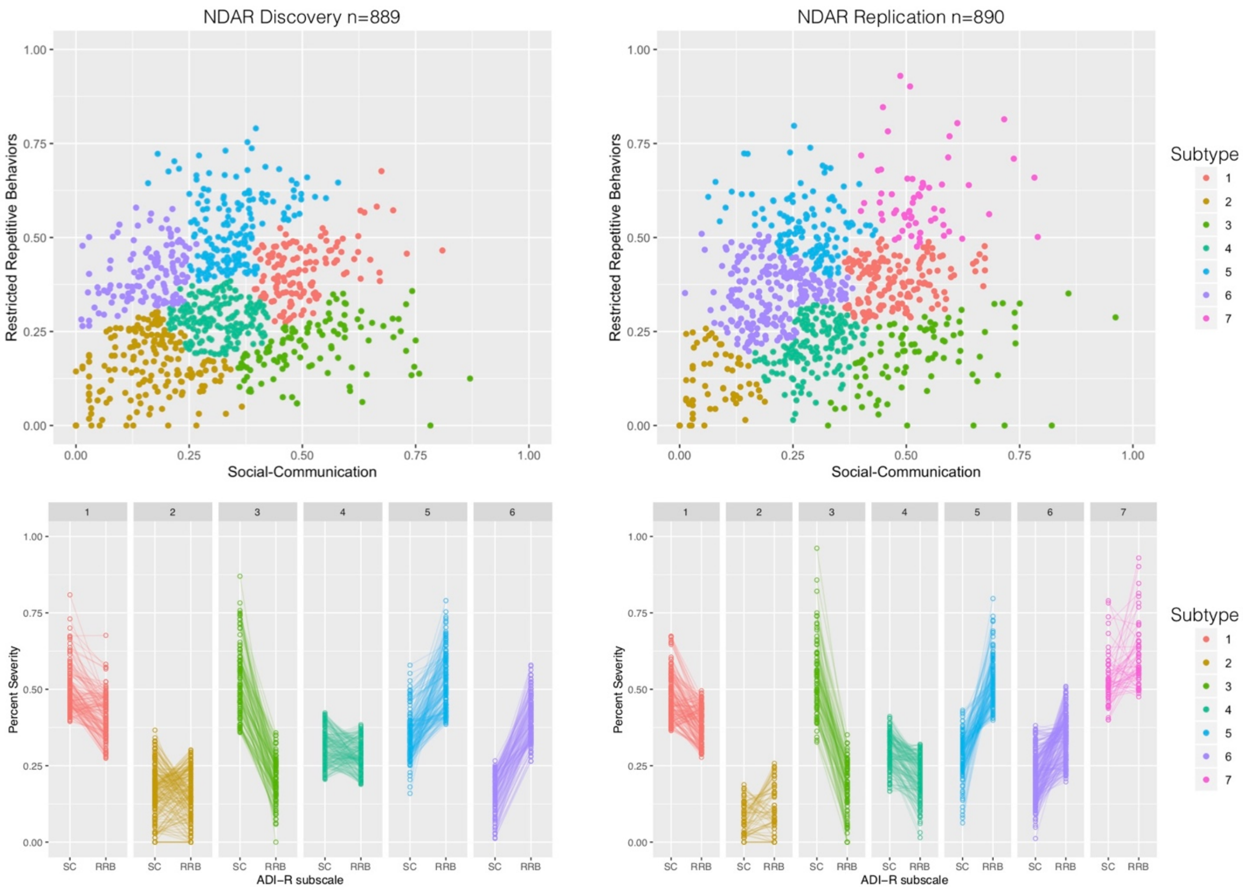

Supplementary Figure 2: Unsupervised stratification with agglomerative hierarchical clustering and the dynamic hybrid tree cut algorithm for identifying clusters. *This figure shows subtypes that emerge from clustering when the number of clusters is automatically selected with a dynamic hybrid tree cutting algorithm.*

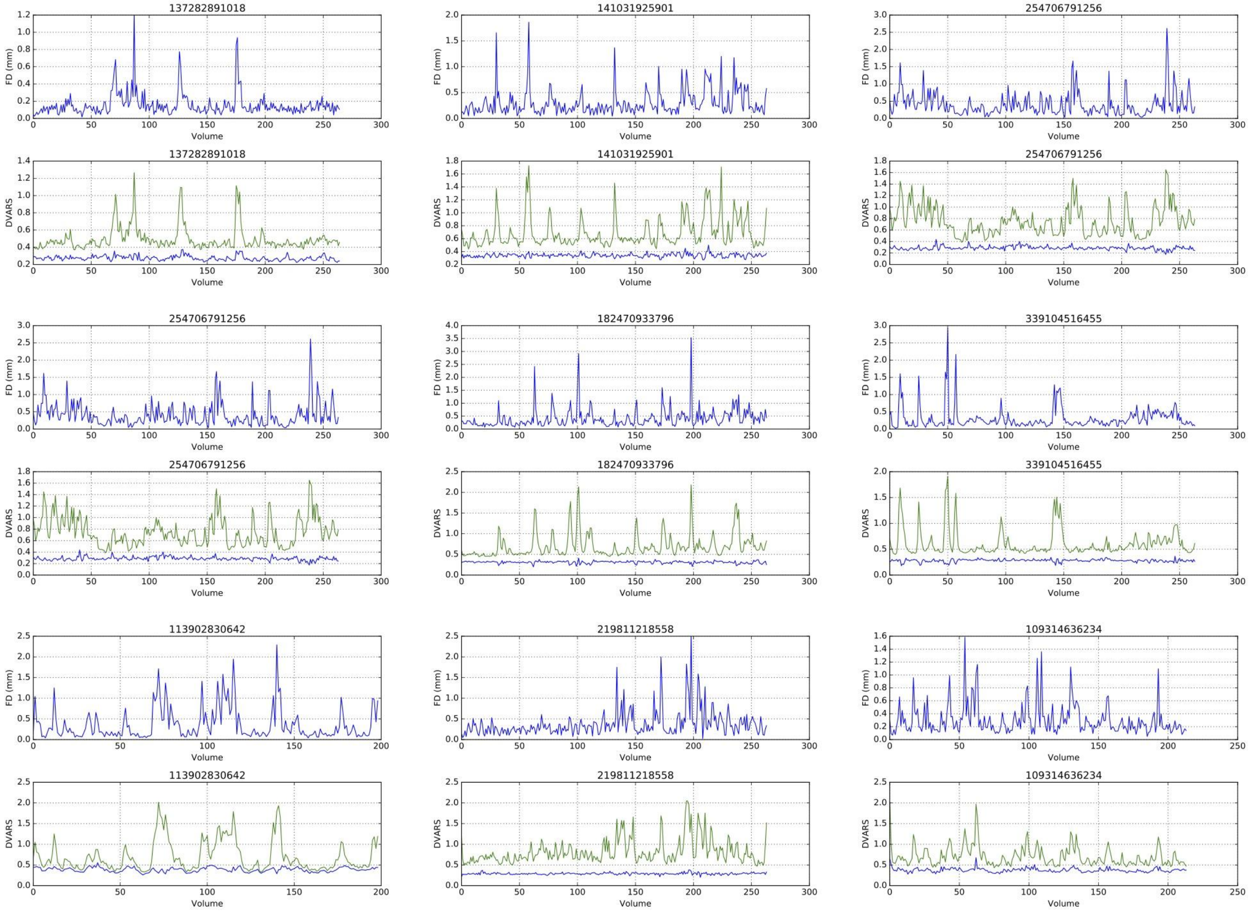

Supplementary Figure 3: Examples of head-motion derived artefact that the effect of ME-ICA at flattening DVARS. *This figure shows 9 example subjects, each with two plots. The top plot always shows framewise displacement (in mm) as an indicator of how much head motion is exhibited at each successive volume. The bottom plot shows DVARS traces from the optimally combined time-series before ME-ICA denoising (green) and after ME-ICA denoising (blue). DVARS traces before ME-ICA (green) closely follow the same pattern of framewise displacement and showcases how head motion can induce non-BOLD artefact. However, after ME-ICA denoising these DVARS traces are heavily flattened out, as a large proportion of this non-BOLD head motion artefact is isolated and removed as part of the denoising process.*

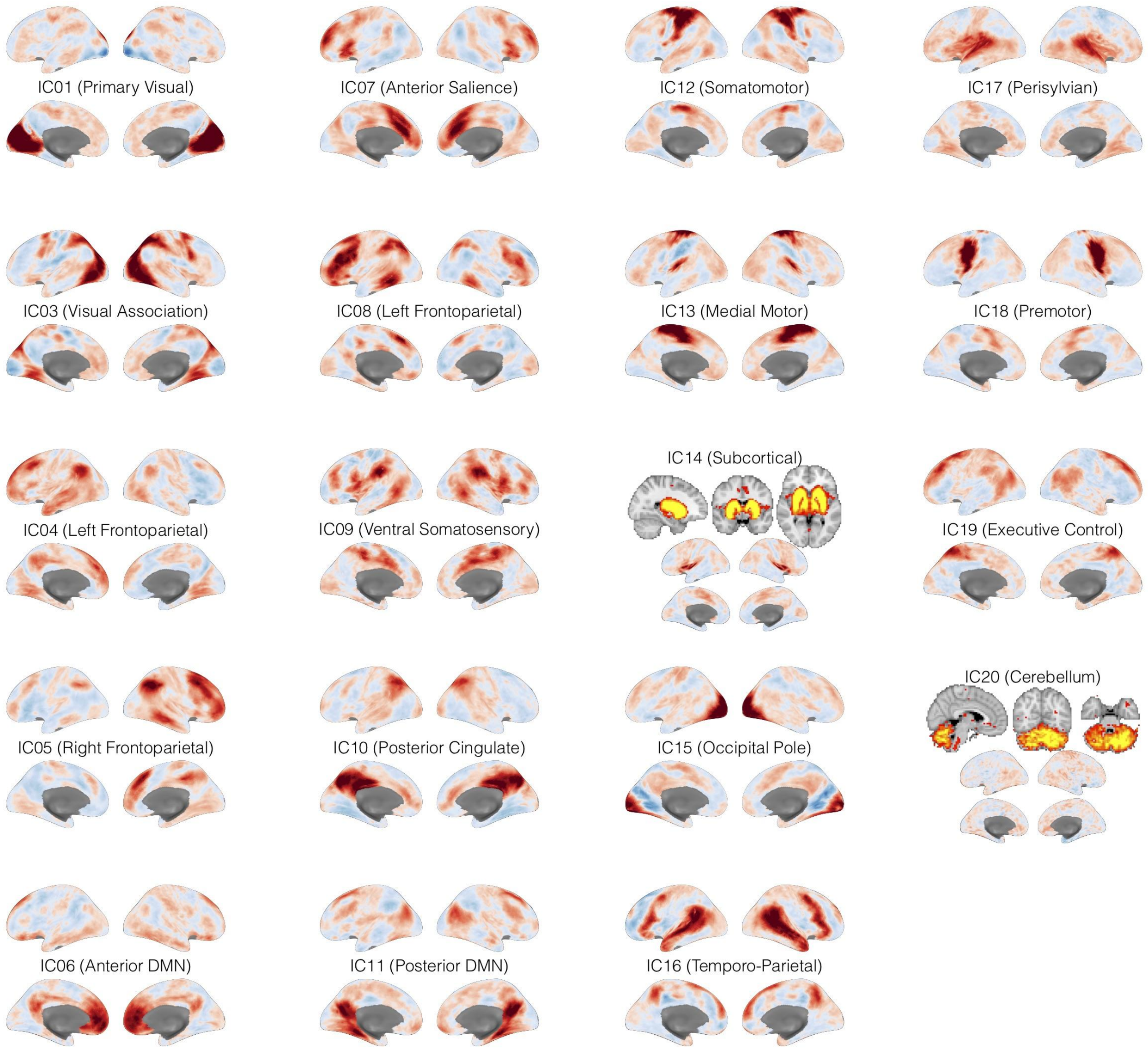

Supplementary Figure 4: Visual depiction of the 19 components identified with group-ICA. *Z-statistics are indicated with the blue-to-red color scale. Increasingly dark red regions are the regions of primary importance for the component. For components with mostly subcortical or cerebellar regions of importance (i.e. IC14 and IC20), these regions are highlighted in bright orange in axial, sagittal, and coronal planes.*

Supplementary Tables

Supplementary Table 1: ADI-R items to use in DSM-5 scoring. *This scoring scheme is identical to that reported by Huerta et al.,*[1]*. Subscales A1-A3 are within the social-communication (SC) domain while subscales B1-B4 are within the restricted repetitive behavior (RRB) domain. All items in A1-A3 utilize the Current scores, while all items in B1-B4 utilize the Ever scores.*

	**< 4 years**	**4 - 10 years**	**> 10 years**	**Description**
**A1**	*COM34,*	*COM34,*	*COM34,*	*Social verbalization and chat,*
	COM35,	*COM35,*	*COM35,*	*Reciprocal conversation,*
	COM31,	*COM31,*	*COM31,*	*Use of other's body to communicate,*
	SOCIAL61,	*SOCIAL61,*		*Imitative social play,*
	SOCIAL52,	*SOCIAL52,*	*SOCIAL52,*	*Showing and directing attention,*
	SOCIAL54,	*SOCIAL54,*	*SOCIAL54,*	*Seeking to share his/her enjoyment with others,*
	SOCIAL55,	*SOCIAL55,*	*SOCIAL55,*	*Offering comfort,*
	COM46,			*Attention to voice,*
	SOCIAL51	*SOCIAL51*	*SOCIAL51*	*Social smiling*
**A2**	*SOCIAL50,*			*Direct gaze,*
	COM42,	*COM42,*	*COM42,*	*Pointing to express interest,*
	COM43,	*COM43,*	*COM43,*	*Nodding,*
	COM44,	*COM44,*	*COM44,*	*Head shaking,*
	COM45,	*COM45,*	*COM45,*	*Conventional/instrumental gestures,*
	SOCIAL57,	*SOCIAL57,*	*SOCIAL57,*	*Range of facial expressions to communicate,*
	SOCIAL56	*SOCIAL56*	*SOCIAL56*	*Quality of social overtures*
**A3**	*COM36,*	*COM36,*	*COM36,*	*Inappropriate questions or statements,*
	SOCIAL58,	*SOCIAL58,*	*SOCIAL58,*	*Inappropriate facial expressions,*
	SOCIAL53,	*SOCIAL53,*	*SOCIAL53,*	*Offering to share,*
	SOCIAL59,	*SOCIAL59,*	*SOCIAL59,*	*Appropriateness of social responses,*
	SOCIAL62,	*SOCIAL62,*		*Interest in children,*
	SOCIAL63,	*SOCIAL63,*		*Responses to approaches of other children,*
		COM49,		*Imaginative play with peers,*
		SOCIAL64,		*Group play with peers,*
		SOCIAL65,	*SOCIAL65,*	*Friendships,*
		SOCIAL66	*SOCIAL66*	*Social disinhibition*
**B1**	*COM33,*	*COM33,*	*COM33,*	*Stereotyped utterances and delayed echolalia,*
	COM37,	*COM37,*	*COM37,*	*Pronominal reversal,*
	COM38,	*COM38,*	*COM38,*	*Neologisms/idiosyncratic language,*
	RRB69,	*RRB69,*	*RRB69,*	*Repetitive use of objects or interest in parts of objects,*
	RRB77,	*RRB77,*	*RRB77,*	*Hand and finger mannerisms,*
	RRB78	*RRB78*	*RRB78*	*Other complex mannerisms or stereotyped body movements*
**B2**	*COM39,*	*COM39,*	*COM39,*	*Verbal rituals,*
	RRB70,	*RRB70,*	*RRB70,*	*Compulsions/rituals,*
	RRB74,	*RRB74,*	*RRB74,*	*Difficulties w/ minor changes in routines or personal environment*
	RRB75	*RRB75*	*RRB75*	*Resistance to trivial changes in the environment (not directly affecting the subject)*

**B3**	*RRB67,<br>RRB68,<br>RRB76*	*RRB67,<br>RRB68,<br>RRB76*	*RRB67,<br>RRB68,<br>RRB76*	*Unusual preoccupations,<br>Circumscribed interests,<br>Unusual attachment to objects*
**B4**	*RRB72,<br>RRB73,<br><br>RRB71*	*RRB72,<br>RRB73,<br><br>RRB71*	*RRB72,<br>RRB73,<br><br>RRB71*	*Undue general sensitivity to noise,<br>Abnormal idiosyncratic negative response to specific sensory stimuli,<br>Unusual sensory interests*

Supplementary Table 2: Ethics approval information for different sites in the EU-AIMS LEAP study.

Site	*Ethics Committee*	*ID/reference no.*
Kings College London, Cambridge	*London-Central and Queen Square Health Research Authority Research Ethics Committee*	*13/LO/1156*
Radboud, Utrecht	*Radboud universitair medisch centrum Instituut Waarborging Kwaliteit en Veiligheid Commissie Mensgebonden Onderzoek Regio Arnhem-Nijmegen (Radboud University Medical Centre Institute Ensuring Quality and Safety Committee on Research Involving Human Subjects Arnhem-Nijmegen)*	*2013/455*
Mannheim	*UMM Universitätsmedizin Mannheim, Medizinische Ethik Kommission II (UMM University Medical Mannheim, Medical Ethics Commission II)*	*2014-540N-MA*

Supplementary References

1 Huerta M, Bishop SL, Duncan A, Hus V, Lord C. Application of DSM-5 criteria for autism spectrum disorder to three samples of children with DSM-IV diagnoses of pervasive developmental disorders. *Am J Psychiatry* 2012; **169**: 1056–1064.
