# Supplementary material for: Imbalanced social-communicative and restricted repetitive behavior subtypes of autism spectrum disorder exhibit different neural circuitry
Source: Commun Biol. 2021 May 14;4:574. doi: 10.1038/s42003-021-02015-2 (PMC8121854; doi:10.1038/s42003-021-02015-2)
Supplement: Supplementary file 2 — Description of Additional Supplementary Files [file 42003_2021_2015_MOESM2_ESM.pdf]

Description of Additional Supplementary Files

File name: Supplementary Data 1

Description: Source data for Figure 2.

File name: Supplementary Data 2

Description: Statistics from all functional connectivity comparisons.

File name: Supplementary Data 3

Description: Gene lists used for enrichment analyses.
