# Supplementary material for: Imbalanced social-communicative and restricted repetitive behavior subtypes of autism spectrum disorder exhibit different neural circuitry
Source: Commun Biol. 2021 May 14;4:574. doi: 10.1038/s42003-021-02015-2 (PMC8121854; doi:10.1038/s42003-021-02015-2)
Supplement: Supplementary file 6 — Reporting Summary [file 42003_2021_2015_MOESM6_ESM.pdf]

# nature research

Corresponding author(s): Michael V. Lombardo

Last updated by author(s): Mar 2, 2021

# Reporting Summary

Nature Research wishes to improve the reproducibility of the work that we publish. This form provides structure for consistency and transparency in reporting. For further information on Nature Research policies, see our Editorial Policies and the Editorial Policy Checklist.

## Statistics

For all statistical analyses, confirm that the following items are present in the figure legend, table legend, main text, or Methods section.

| n/a | Confirmed | |
|---|---|---|
| ☐ | ☒ | The exact sample size (*n*) for each experimental group/condition, given as a discrete number and unit of measurement |
| ☐ | ☒ | A statement on whether measurements were taken from distinct samples or whether the same sample was measured repeatedly |
| ☐ | ☒ | The statistical test(s) used AND whether they are one- or two-sided<br>*Only common tests should be described solely by name; describe more complex techniques in the Methods section.* |
| ☐ | ☒ | A description of all covariates tested |
| ☐ | ☒ | A description of any assumptions or corrections, such as tests of normality and adjustment for multiple comparisons |
| ☐ | ☒ | A full description of the statistical parameters including central tendency (e.g. means) or other basic estimates (e.g. regression coefficient) AND variation (e.g. standard deviation) or associated estimates of uncertainty (e.g. confidence intervals) |
| ☐ | ☒ | For null hypothesis testing, the test statistic (e.g. *F*, *t*, *r*) with confidence intervals, effect sizes, degrees of freedom and *P* value noted<br>*Give P values as exact values whenever suitable.* |
| ☒ | ☐ | For Bayesian analysis, information on the choice of priors and Markov chain Monte Carlo settings |
| ☒ | ☐ | For hierarchical and complex designs, identification of the appropriate level for tests and full reporting of outcomes |
| ☐ | ☒ | Estimates of effect sizes (e.g. Cohen's *d*, Pearson's *r*), indicating how they were calculated |

*Our web collection on statistics for biologists contains articles on many of the points above.*

## Software and code

Policy information about availability of computer code

| | |
|---|---|
| Data collection | N/A |
| Data analysis | Tidy data and reproducible analysis code for this study is available at https://github.com/IIT-LAND/adir_subtyping. |

For manuscripts utilizing custom algorithms or software that are central to the research but not yet described in published literature, software must be made available to editors and reviewers. We strongly encourage code deposition in a community repository (e.g. GitHub). See the Nature Research guidelines for submitting code & software for further information.

## Data

Policy information about availability of data

All manuscripts must include a data availability statement. This statement should provide the following information, where applicable:

- Accession codes, unique identifiers, or web links for publicly available datasets
- A list of figures that have associated raw data
- A description of any restrictions on data availability

Tidy data and reproducible analysis code for this study is available at https://github.com/IIT-LAND/adir_subtyping.

# Field-specific reporting

# Life sciences study design

All studies must disclose on these points even when the disclosure is negative.

| | |
|---|---|
| Sample size | Sample size calculations were not performed for the NDAR dataset, because this was already pre-existing data and we utilized all available ADI-R data within NDAR. On December 13, 2019 we conducted a search of NDAR to extract all datasets utilizing the ADI-R. This resulted in 60 independent datasets totaling 2,628 unique individuals. From here, we filtered for all individuals who had data for the verbal communication items (e.g., acquisition of words, phrases, social verbalization, chit-chat, reciprocal conversation), leaving a total of 1,781 individuals across 57 independent datasets.<br><br>Power and sample size calculations for the EU-AIMS LEAP were provided in a previous paper by Loth et al., (2017). Please see Additional File 3, which accompanies this previous paper for power and sample size calculations - https://bit.ly/39vy8yK.<br><br>Loth, E., Charman, T., Mason, L. et al. The EU-AIMS Longitudinal European Autism Project (LEAP): design and methodologies to identify and validate stratification biomarkers for autism spectrum disorders. Molecular Autism 8, 24 (2017). https://doi.org/10.1186/s13229-017-0146-8 |
| Data exclusions | No data was excluded from the NDAR dataset.<br><br>For EU-AIMS LEAP, we selected all participants for whom structural and rsfMRI data were available. However, n=120 participants had to be excluded from the analysis because of missing ADI-R item-level data (n=64), missing IQ data (n=3), or because preprocessing could not be completed for a variety of reasons (e.g., registration/normalization errors because of poor quality MPRAGE data, poor anatomical coverage, or large anatomical deviance such as large ventricles (n=39), incomplete rsfMRI data (n=3), errors in convergence of the ME-ICA algorithm (n=11)). |
| Replication | From the NDAR dataset we split the data into independent Discovery and Replication sets. From the total of 1,781 individuals across 57 independent datasets, we took each of these 57 datasets and randomly split the dataset in half. This achieved a half split for independent Discovery and Replication sets that are balanced across the 57 datasets and by sex (Discovery n=889, mean age = 8.91 years, SD age = 5.26 years, 77% male; Replication n=890, mean age = 8.89 years, SD age = 5.37 years, 77% male).<br><br>For the EU-AIMS LEAP dataset, the final sample size was n=266 autistic and n=243 TD participants. This final sample was split into independent Discovery and Replication sets (balanced for sex and age) for the purpose of identifying functional connectivity differences that are replicable. |
| Randomization | No randomization was done. |
| Blinding | No blinding was done. |

# Reporting for specific materials, systems and methods

We require information from authors about some types of materials, experimental systems and methods used in many studies. Here, indicate whether each material, system or method listed is relevant to your study. If you are not sure if a list item applies to your research, read the appropriate section before selecting a response.

## Materials & experimental systems

| n/a | Involved in the study | |
|---|---|---|
| x | | Antibodies |
| x | | Eukaryotic cell lines |
| x | | Palaeontology and archaeology |
| x | | Animals and other organisms |
| | x | Human research participants |
| x | | Clinical data |
| x | | Dual use research of concern |

## Methods

| n/a | Involved in the study | |
|---|---|---|
| x | | ChIP-seq |
| x | | Flow cytometry |
| | x | MRI-based neuroimaging |

# Human research participants

Policy information about studies involving human research participants

| | |
|---|---|
| Population characteristics | A full characterization of the NDAR and EU-AIMS LEAP datasets can be found in Tables 1 and 2 of the manuscript. |
| Recruitment | The EU-AIMS LEAP data comes from a large multisite European initiative with the aim of identifying biomarkers for ASD. EU-AIMS LEAP recruited 437 individuals with ASD and 300 TD individuals, both male and female, aged between 6 and 30 years. Participants underwent comprehensive clinical, cognitive, and MRI assessment at one of the following five centers: Institute of Psychiatry, Psychology and Neuroscience, King's College London, United Kingdom; Autism Research Centre, University of Cambridge, United Kingdom; Radboud University Nijmegen Medical Centre, the Netherlands; University Medical Centre Utrecht, the Netherlands; and Central Institute of Mental Health, Mannheim, Germany. For further details about the study design, we refer to Loth et al., (2017), and for a comprehensive clinical characterization of the LEAP cohort, we refer to Charman et al., (2017).<br><br>Loth, E., Charman, T., Mason, L. et al. The EU-AIMS Longitudinal European Autism Project (LEAP): design and methodologies to identify and validate stratification biomarkers for autism spectrum disorders. Molecular Autism 8, 24 (2017). https://doi.org/10.1186/s13229-017-0146-8<br><br>Charman, T., Loth, E., Tillmann, J. et al. The EU-AIMS Longitudinal European Autism Project (LEAP): clinical characterisation. Molecular Autism 8, 27 (2017). https://doi.org/10.1186/s13229-017-0145-9 |
| Ethics oversight | The study was approved by the local ethical committees of participating centers (see Supplementary Table 2 of the manuscript for information about the ethics committees), and written informed consent was obtained from all participants or their legal guardians (for participants <18 years). |

Note that full information on the approval of the study protocol must also be provided in the manuscript.

# Magnetic resonance imaging

## Experimental design

| | |
|---|---|
| Design type | Resting state fMRI |
| Design specifications | Participants were instructed to relax, with eyes open and fixate on a cross presented on the screen for the duration of the rsfMRI scan. |
| Behavioral performance measures | N/A |

## Acquisition

| | |
|---|---|
| Imaging type(s) | Functional MRI |
| Field strength | 3T |
| Sequence & imaging parameters | An eight-to-ten minute resting-state fMRI (rsfMRI) scan was acquired using a multi-echo planar imaging (ME-EPI) sequence; TR=2300ms, TE~12ms, 31ms, and 48ms (slight variations are present across centers), flip angle=80°, matrix size=64x64, in-plane resolution=3.8mm, FOV=240mm, 33 axial slices, slice thickness/gap=3.8mm/0.4mm, volumes=200 (UMCU), 215 (KCL, CIMH), or 265 (RUMC, UCAM). |
| Area of acquisition | Whole-brain imaging |

Diffusion MRI ☐ Used ☒ Not used

## Preprocessing

| | |
|---|---|
| Preprocessing software | Multi-echo rsfMRI data were preprocessed with the multi-echo independent components analysis (ME-ICA) pipeline, implemented with the meica python library (v3.2) (https://github.com/ME-ICA/me-ica). |
| Normalization | Non-linear warping achieved using AFNI's 3dQWarp. |
| Normalization template | MNI template |
| Noise and artifact removal | Multi-echo ICA was used as the denoising procedure. |
| Volume censoring | No volume censoring was done. |

## Statistical modeling & inference

| | |
|---|---|
| Model type and settings | Group-ICA, dual regression, followed by between-components connectivity analysis based on partial correlations estimated with FSLNets (ridge regression). Models implementing the main hypothesis tests of subtype differences were computed as linear mixed effect models (lme function from the nlme library in R), whereby connectivity was the dependent variable, and |

subtype, sex, and age were used as fixed effect independent variables and site was modeled with random intercepts as a random effect. These models were computed separately for the Discovery and Replication set. Connectivity pairs were deemed as showing replicable subtype differences if the Discovery set showed an effect at $p<0.05$ and the replication Bayes Factor statistic computed on t-statistics from Discovery and Replication sets was greater than 10 (repBF>10), indicating strong evidence in favor of replication.

Effect(s) tested

SC-RRB balance subtypes were tested against the TD group and also against each other.

Specify type of analysis: ☒ Whole brain ☐ ROI-based ☐ Both

Statistic type for inference
(See Eklund et al. 2016)

Not applicable as we used replication Bayes Factor tests to test whether effects identified in a Discovery set replicated in an independent Replication set.

Correction

Not applicable as we used replication Bayes Factor tests to test whether effects identified in a Discovery set replicated in an independent Replication set.

## Models & analysis

| n/a | Involved in the study |
|---|---|
| ☐ | ☒ Functional and/or effective connectivity |
| ☒ | ☐ Graph analysis |
| ☒ | ☐ Multivariate modeling or predictive analysis |

Functional and/or effective connectivity

Time courses for each subject and each independent component (IC) were used to model between-component connectivity. This was achieved by constructing a partial correlation matrix amongst all 19 components using Tikhonov-regularization (i.e. ridge regression, rho=1) as implemented within the nets_netmats.m function in the FSLNets MATLAB toolbox (https://fsl.fmrib.ox.ac.uk/fsl/fslwiki/FSLNets). The aim of utilizing partial correlations was to estimate direct connection strengths in a more accurate manner than can be achieved with full correlations, which allow more for indirect connections to influence connectivity strength. Partial correlations were then converted into Z-statistics using Fisher's transformation for further statistical analyses. The lower diagonal of each subject's partial correlation matrix was extracted for a total of 171 separate component-pair comparisons.
